# Reply to Gross et al.: Indirect reciprocity undermines large-scale cooperation under realistic conditions

**DOI:** 10.1073/pnas.2410085121

**Published:** 2024-06-24

**Authors:** Eric Schnell, Michael Muthukrishna

**Affiliations:** ^a^Department of Psychological and Behavioural Science, London School of Economics and Political Science, London WC2A 2AE, United Kingdom

Gross et al. (1) point out that in our model of how “indirect reciprocity undermines indirect reciprocity destabilizing large-scale cooperation” (2), we do not consider intergroup interactions between players in different local groups in the Mutual Aid Game (MAG). This was by design. As we say in the paper, “if group members interact more frequently across local group boundaries and move between groups, the effective population becomes closer to the global population incentivizing higher-scale cooperation (3, 4).” Our model is based on the assumption that local subgroups do indeed exist, and by definition, these local groups are the people that we interact with more frequently and to which we show a preference for cooperation—ingroup favoritism (5, 6). If individuals were to interact with a large number of outgroup members more frequently than they do ingroup members, then we agree that global cooperation would be more stable. In fact, we argue that one the keys to building large-scale cooperation is to breakdown the mechanisms that incentivize ingroup biases (7, 8). However, there is a problem when mapping from our models to the real world. Whether people would interact and cooperate more with those in their local ingroup than outgroup members is precisely what we are trying to explain.

**Fig. 1.**
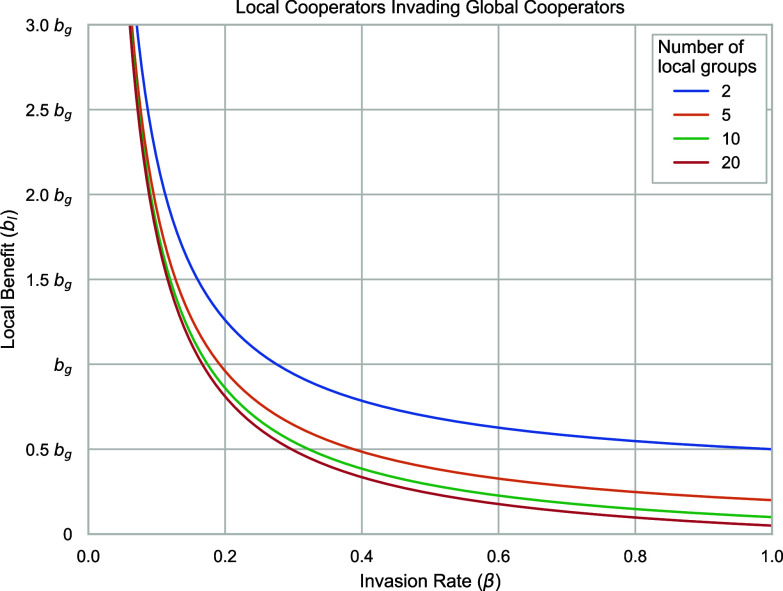
Minimum benefit of local cooperation ($b_{l}$), in relation to the benefit of global cooperation ($b_{g}$), required for local cooperators to invade global cooperators, given an invasion rate (*β*) and number of local groups (*a*). For $b_{l}<b_{g}$, the benefits of global cooperation outweigh those of local cooperation, and so any value below this point means that local cooperators invade global cooperators even when potential global returns outweigh potential local returns. We see how no matter how many local groups there are, local cooperators can outcompete and invade global cooperators with a minority of invaders, even when global cooperation is favorable. For more details, see ref. 2.

Our paper shows that even when the potential payoff for large-scale global cooperation is higher than smaller-scale cooperation, smaller-scale local cooperation persists because indirect reciprocity is insufficient to prevent large-scale free-riding. Moreover, as figure 4 of our paper (Fig. 1 below) reveals, local cooperation undermines global cooperation, even when local cooperators are receiving a larger benefit from the global Public Goods Game (PGG). They are effectively free riding on the global PGG while still receiving aid from their local interactions. In our model, we see that so long as roughly a quarter of MAG interactions are with like-minded peers; local cooperators can invade global cooperators even when global cooperation provides higher returns. This does not represent exactly the same process as if the MAG included intergroup interactions, but it does show that local cooperation can undermine global cooperation even in groups where the majority of members favor global cooperation.

Indeed, Gross et al.’s paper on the evolution of universal cooperation also reveals the pervasiveness of local groups in eroding universal cooperation (3). Universal cooperation only becomes viable as groups become more interconnected (best illustrated in figure 2 of their paper). When players interact with ingroup members more than they do outgroup members, which is definitionally and empirically more realistic, then they also find that local cooperation undermines global cooperation. Our model and Gross et al.’s model are complementary. Any disagreement is whether it is realistic to assume that people will interact with their local communities—those they live, work, and socialize with—more than they interact with the vast majority of people in their counties, states, and countries. On the basis of both models, we argue that it is fair to say that indirect reciprocity undermines large-scale cooperation under realistic conditions.
